# New insights into developmental fate decisions by autoreactive CD4 thymocytes

**DOI:** 10.1002/ctm2.1590

**Published:** 2024-02-16

**Authors:** Xuguang Tai, Alfred Singer

**Affiliations:** ^1^ Experimental Immunology Branch, National Cancer Institute National Institutes of Health Bethesda Maryland USA

The primary function of the thymus is to select major histocompatibility complex (MHC)‐restricted T cells for immune responses against pathogenic infections and to simultaneously establish self‐tolerance to avoid autoimmunity. These processes are mediated by the interaction of T‐cell receptor (TCR) expressed on developing thymocytes with the self‐peptide MHC ligands expressed in the thymus.^1^, ^2^ Thymocytes are initially signalled by TCR ligands present in the thymic cortex to undergo positive selection and then migrate to the thymic medulla for negative selection.^3^, ^4^ During positive selection, TCR signalling upregulates anti‐apoptotic protein Bcl‐2 to promote the survival of developing thymocytes, whereas during negative selection, thymocytes with potentially autoreactive TCRs are agonist signalled and prevented from continuing their maturation by undergoing clonal deletion or, alternatively, by differentiating into either Foxp3^+^Tregs or IL‐2^+^Teffs.

We recently assessed the timing of clonal deletion, Treg differentiation and Teff differentiation, and found that clonal deletion occurred early after positive selection, whereas Foxp3^+^Tregs and IL‐2^+^Teffs arose later. We then determined whether these three fates were signalled simultaneously or sequentially during thymic selection, as it was possible that all three fates were signalled early but that thymocytes died faster than they expressed Foxp3 or interleukin‐2 (IL‐2). To assess this possibility, we generated a new experimental mouse (called ZAP70^TgKO^), in which ZAP70 expression was turned off upon positive selection, so that thymocytes could only transduce agonist signals early but not late in CD4^+^ T‐cell differentiation.^5^ These studies demonstrate for the first time that clonal deletion and autoreactive T‐cell differentiation are agonist signalled at different times during thymic selection with early agonist signals inducing clonal deletion and late agonist signals inducing surviving autoreactive thymocytes to differentiate into either Tregs or Teffs (Figure 1).

**FIGURE 1:**
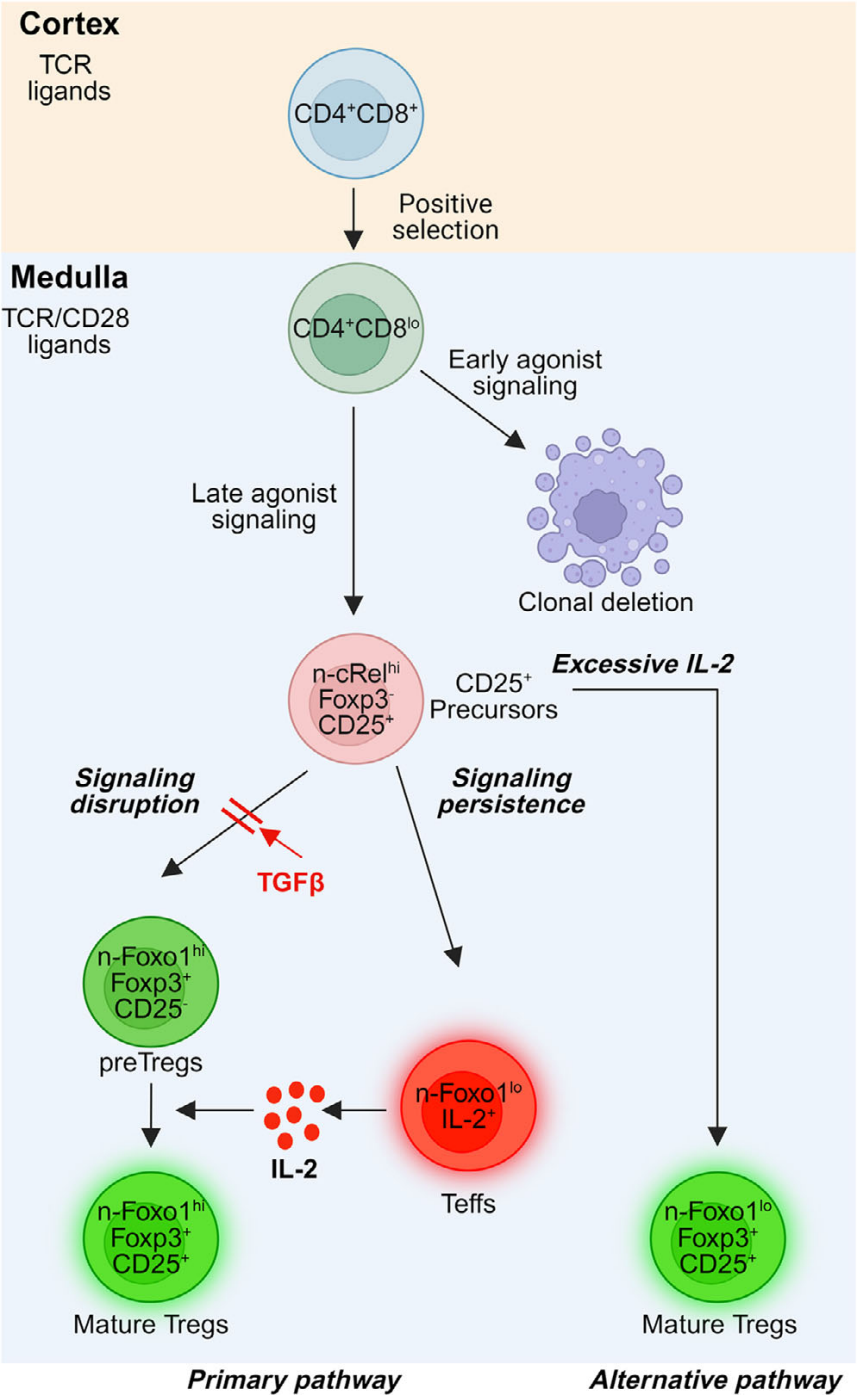
Schematic illustration of autoreactive CD4 thymocyte development in the thymus (created using biorender.com).

We then assessed the fate of autoreactive CD4 thymocytes that avoided clonal deletion. Surviving cells that receive late agonist signalling become CD4^+^CD25^+^ precursors for both Foxp3^+^Tregs and IL‐2^+^Teffs.^6^ However, it is not clear how CD4^+^CD25^+^ precursors determine their appropriate Treg or Teff cell fate. Our previous in vitro studies showed that CD28 costimulation induces Foxp3 expression to initiate Treg differentiation. Importantly, however, induction of Foxp3 expression requires cessation of agonist stimulation,^7^ which implies that agonist signalling and its disruption are both required to induce Foxp3 gene expression. Indeed, our recent studies confirmed that physically disrupting in vivo agonist signalling induced Treg differentiation. Mechanistically, we found that agonist signalling first upregulated cRel, the pioneer transcription factor for Treg differentiation, while disruption of agonist signalling subsequently upregulated nuclear Foxo1 (n‐Foxo1) whose expression is also indispensable for Foxp3 transcription. The sequential induction of the two transcription factors cRel and Foxo1 induced CD4^+^CD25^+^ precursors to express Foxp3 and to differentiate into Foxp3^+^CD25^−^ preTregs, which then became Foxp3^+^CD25^+^ mature Tregs upon IL‐2 signalling. In contrast to Treg differentiation, which requires agonist signalling disruption, we found that CD4^+^CD25^+^ precursors require persistent agonist signalling to activate the IL‐2 gene and to become IL‐2^+^Teffs.^5^ Thus, the fate of surviving CD4 thymocytes depends on the duration of late agonist signalling, with disrupted agonist signalling inducing Foxp3^+^Tregs and persistent agonist signalling inducing IL‐2^+^Teffs (Figure 1).

Because transforming growth factor beta (TGFβ) has previously been shown to induce Tregs through unknown mechanisms,^8^, ^9^ our finding that late agonist signalling disruption was required to induce Foxp3 gene expression prompted us to examine whether TGFβ functions by disrupting agonist signalling in autoreactive CD4 thymocytes. Indeed, we found that TGFβ promotes Foxp3 gene expression and thymic Treg development by specifically disrupting in vivo agonist signalling to promote n‐Foxo1 expression.^5^ This finding provides a novel mechanism for how TGFβ regulates thymic Treg differentiation. Notably, our studies show that TGFβ disrupts in vivo agonist signalling in a TCR dose‐dependent manner, such that TGFβ is only present in vivo in amounts sufficient to disrupt weaker, but not stronger, TCR/CD28 agonist signalling.^5^ As a result, weaker TCR/CD28 agonist signalling is disrupted in vivo and induces differentiation into Tregs, whereas stronger TCR/CD28 agonist signalling persists in vivo and induces CD4^+^CD25^+^ precursors to differentiate into IL‐2^+^Teff cells (Figure 1). These findings provide a straightforward explanation for why different autoreactive development fates are correlated with TCR affinities as indicated by the affinity hierarchy: clonal deletion > Teffs > Tregs, and for why there are always less Teffs than Tregs in the thymus.^5^ We think there is a narrow window of TCR affinity for IL‐2^+^Teff differentiation, and this window is expanded in the absence of TGFβ signalling, where low affinity TCR signalling persists and diverts CD4^+^CD25^+^ precursors into Teffs instead of Tregs. Consequently, excessive IL‐2 is produced in the TGFβR1‐deficient mice.^5^

Our discovery that CD4^+^CD25^+^ precursors first become Foxp3^+^CD25^−^ preTregs and then Foxp3^+^CD25^+^ mature Tregs defines a precursor–progeny relationship that reveals a new Treg developmental pathway, which we have called the ‘primary pathway’ (Figure 1). This finding contradicts the conventional perspective that IL‐2 directly promotes CD4^+^CD25^+^ precursors to become Foxp3^+^CD25^+^ mature Tregs (which we have called the alternative pathway).^10^, ^11^ In fact, only excessive non‐physiologic amounts of in vivo IL‐2 induced CD4^+^CD25^+^ precursors to express Foxp3, and this occurs because of their high expression of ThPOK‐induced SOCS1 that impairs IL‐2 signal transduction.^5^, ^12^ However, under physiological conditions, the amount of IL‐2 present is limited by Teff cell numbers and is insufficient to activate the IL‐2‐induced alternative pathway.^5^, ^6^, ^11^ Notably, IL‐2 itself was not needed to induce Foxp3 expression, but it was required to promote the survival of Foxp3^+^CD25^−^ preTreg and their differentiation into Foxp3^+^CD25^+^ mature Tregs.^5^, ^11^ The key difference between the primary pathway and the alternative pathway is whether CD4^+^CD25^+^ precursors pass through Foxp3^+^CD25^−^ preTreg stage to become Foxp3^+^CD25^+^ mature Tregs (Figure 1). Since most Foxp3^+^CD25^−^ preTregs die by Foxp3‐induced apoptosis in normal mice,^11^ their existence as immediate Treg precursors has been largely overlooked. Consequently, we propose that thymic Treg differentiation goes through the primary pathway because this pathway is TGFβ dependent and occurs in normal mice.

Overall, our analysis of autoreactive CD4 thymocyte differentiation revealed an unexpected similarity with normal thymocyte development described by the kinetic signalling model,^2^, ^13^, ^14^ as signalling disruption versus signalling persistence is the underlying mechanism of lineage fate determination in both. It remains to be determined how TGFβ disrupts agonist TCR signalling, and whether TGFβ plays additional roles for Treg differentiation aside from disruption of agonist signalling.

## AUTHOR CONTRIBUTIONS

Not Applicable.

## CONFLICT OF INTERESTS STATEMENT

The authors declare no conflict of interest.

## ETHICS STATEMENT

Not Applicable.
